# *Serratia marcescens* antibiotic resistance mechanisms of an opportunistic pathogen: a literature review

**DOI:** 10.7717/peerj.14399

**Published:** 2023-01-05

**Authors:** Faviola Tavares-Carreon, Karla De Anda-Mora, Idalia C. Rojas-Barrera, Angel Andrade

**Affiliations:** 1 Facultad de Ciencias Biológicas, Universidad Autónoma de Nuevo León, Monterrey, Nuevo León, México; 2 Departamento de Microbiología, Facultad de Medicina, Universidad Autónoma de Nuevo León, Monterrey, Nuevo León, México; 3 Environmental Genomics Group, Max Planck Institute for Evolutionary Biology, Plön, Germany; 4 Christian-Albrechts-University Kiel, Kiel, Germany

**Keywords:** *Serratia marcescens*, Opportunistic pathogen, Intrinsic resistance, Acquired resistance, Antimicrobial resistance

## Abstract

*Serratia marcescens* is a ubiquitous bacterium from order *Enterobacterales* displaying a high genetic plasticity that allows it to adapt and persist in multiple niches including soil, water, plants, and nosocomial environments. Recently, *S. marcescens* has gained attention as an emerging pathogen worldwide, provoking infections and outbreaks in debilitated individuals, particularly newborns and patients in intensive care units. *S. marcescens* isolates recovered from clinical settings are frequently described as multidrug resistant. High levels of antibiotic resistance across *Serratia* species are a consequence of the combined activity of intrinsic, acquired, and adaptive resistance elements. In this review, we will discuss recent advances in the understanding of mechanisms guiding resistance in this opportunistic pathogen.

## Introduction

The genus *Serratia* comprises ubiquitous Gram-negative rod-shaped bacteria belonging to the *Enterobacterales* order (Adeolu et al., 2016; Grimont, Grimont & De Rosnay, 1977). So far, 23 species are recognized within this genus, six of which have been associated with human infections, including *S. marcescens*, *S. plymuthica, S. liquefaciens*, *S. rubidaea*, *S. odorifera,* and *S. fonticola.* From these, *S.* *marcescens* is the species most commonly isolated (Traub, 2000). In addition, *S.* *marcescens* can infect a vast set of hosts including corals, insects, nematodes, plants, and mammals (Mahlen, 2011).

*S. marcescens* operates as an opportunistic pathogen mainly affecting patients with prior antibiotic treatment or hosts with a weakened immune system. *S. marcescens* outbreaks are frequently reported in the literature (*e. g.*, over 100 records at the Outbreak database from 1968 to 2019 (Vonberg et al., 2011); however, the majority of infections are likely individual cases (Wisplinghoff et al., 2004). At the nosocomial setting *S. marcescens* infections are more frequently reported in neonatology and intensive care units (ICU), where this microorganism has been isolated from catheters, oxygenation devices, prefilled syringes, needles, parenteral solutions, milk-drawers, sinks, nails, and hands of health care workers. It has been also found in disinfectant solutions or double-distilled water, reflecting its enormous metabolic versatility and capability to adapt and survive in adverse environments (Casolari et al., 2005; Greco-Stewart et al., 2012; Redondo-Bravo et al., 2019).

**Figure 1:**
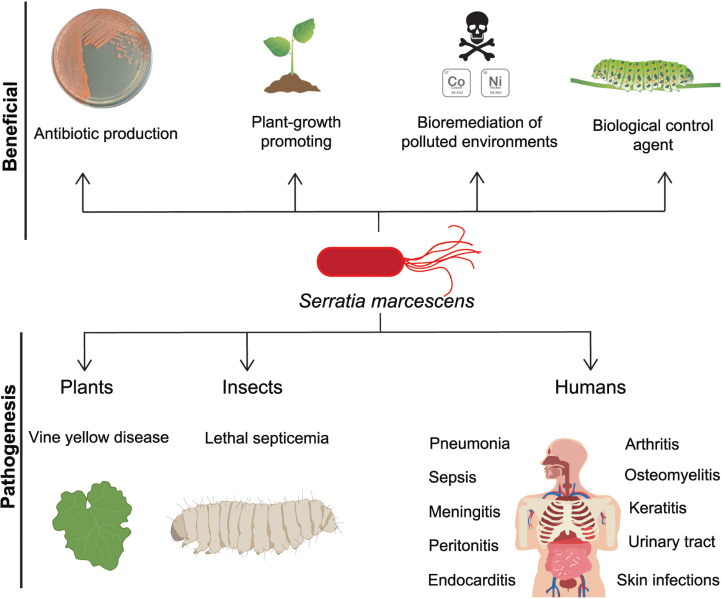
*S. marcescens*, beneficial and pathogenic effects. *S. marcescens* is distributed worldwide and it has positive environmental effects, it can produce antimicrobial compounds such as prodigiosin, as well as factors that promote plant growth. Some member of *Serratia* genus can degrade a wide variety of compounds including chitin and pollutants as heavy metals, therefore they can be considered as biological control agents. In counterpart, a significant phytosanitary risk can be associated to *S. marcescens*, based on its potential to damage several crops, including cucurbits, maize, pepper, sunflower, among others. This bacterium is particularly relevant as an opportunistic human pathogen displaying multi-resistant against several classes of antibiotics.

Within the wide range of infections provoked by *S. marcescens* are found pneumonia, sepsis, meningitis, peritonitis, endocarditis, arthritis, osteomyelitis, keratitis, and urinary tract and skin infections (Fig. 1) (David et al., 2006; Korner et al., 1994; Su et al., 2003; Voelz et al., 2010). Clinical management of *S. marcescens* infections is challenging due to its intrinsic resistance to different classes of antibiotics such as ampicillin, first and second generation cephalosporins, macrolides, and cationic antimicrobial peptides (CAPs) (Ball, McGhie & Geddes, 1977; Evans, Feola & Rapp, 1999). Accordingly, patients in ICU under antibiotic regimen can be considered at higher risk to develop infections by this bacterium.

The infections by *S. marcescens* are commonly treated with cefepime or carbapenems (Siedner et al., 2014; Tamma et al., 2022). In addition, most *S. marcescens* isolates are found susceptible to the aminoglycoside amikacin, however recent reports indicate an increasing resistance to gentamicin and tobramycin (Bertrand & Dowzicky, 2012; Sader et al., 2014). In fact, current emergence of multi-resistant isolates has narrowed the therapeutic options against this pathogen, situation that has been recently alerted by the WHO WHO, 2017.

Bacterial exposure to antibiotics is considered a main factor leading to resistant phenotypes. Nonetheless, a recent analysis of *S. marcescens* isolates responsible for an outbreak in 1969 in a Spanish neonatal intensive care unit revealed the presence of resistance genes toward antibiotics and disinfectants non commercialized by the time of their original isolation (Saralegui et al., 2020). Also, a comparison between strains collected during the 1940s and the beginning of the twenty-first centuries indicated that resistance to different classes of antibiotics already existed in ancient *S. marcescens* isolates (Fuste et al., 2012), pointing out existence of additional factors unrelated to drug therapy driving evolution of such genes.

In this scenario, a recent analysis of 32 *Serratia* spp. genomes led to the conclusion that environmental strains represent an underestimated reservoir for antibiotic resistance determinants (Sandner-Miranda et al., 2018), suggesting that antibiotic resistance genes (ARG) in this genus are inherently encoded. And despite, 76.5% of annotated *Serratia* genomes correspond to clinical isolates, they seem to encode a comparable number of ARG as environmental *Serratia* strains (Sandner-Miranda et al., 2018).

**Figure 2:**
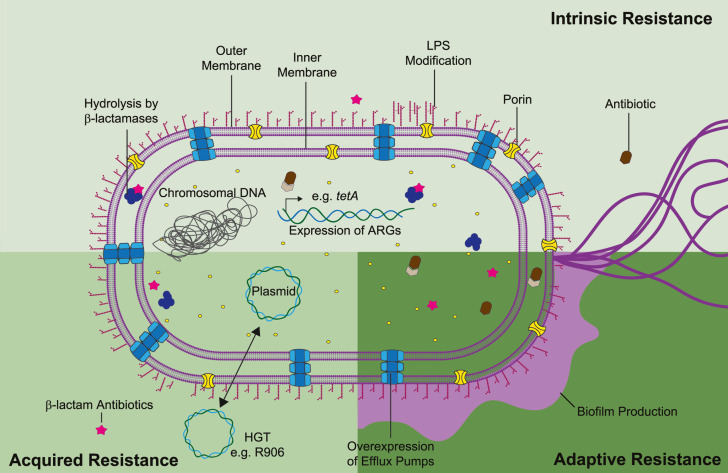
Mechanisms of antibiotic resistance in *S. marcescens* can be divided as intrinsic, acquired, and adaptive resistance. *S. marcescens* intrinsic resistance is related with ARG inherently found in the chromosome (*e.g.*, β-lactamases and efflux pumps), along with lipopolysaccharide (LPS) modifications (pale green rectangle). Acquired resistance is associated with HGT, genetic material transfer between species could derivate in antibiotic resistance and propagation of such phenotype beyond interspecies (lime green square). Adaptive resistance often arises due to pressures from the ecological niche inducing changes in cell permeability and biofilm formation (dark green square). Concerted activity of these mechanisms migth lead in synergistic drug resistance in *S. marcescens*. Each antibiotic resistance mechanisms is discussed in the appropriate subsection of this review.

Antimicrobial resistance mechanisms can be classified as intrinsic, acquired, or adaptive (Fig. 2). Intrinsic resistance refers to the inherent properties of a microorganism that limit the action of antimicrobials *e.g.*, decreased membrane permeability and active efflux of toxic compounds. Acquired resistance might occur due to acquisition of ARG, most commonly through plasmid horizontal gene transfer, or as result of gene mutations. Lastly, adaptive resistance, also called phenotypic resistance, is linked to expeditious transcriptome adjusting in response to stress conditions or environmental stimuli (Hasan, Dutta & Nguyen, 2021). Contrary to intrinsic or acquired resistance, adaptive resistance is a non-inheritable phenotype that generally reverts upon removal of the triggering signal. In *S. marcescens* temperature, pH, oxidative stress, and exposure to disinfectants have been linked with gene regulation and higher bacterial survival (Begic & Worobec, 2008; de Ondarza, 2017; Maseda et al., 2009). Nonetheless, adaptive phenotypes are still modestly understood in the context of antibiotic resistance. Thus, in this review we will focus on the current understanding of intrinsic and acquired mechanisms guiding *S. marcescens* multi-resistance.

## Survey methodology

Primary and secondary literature relevant to the topic of this review was assessed using PubMed (MeSH), MedLine, Google Scholar, and Web of Science using the search terms for articles and their combinations in English to search: “*Serratia*”, “*Serratia marcescens*”, “Entereobacterials”, “*Serratia* infections” in combination with, “diseases outbreak”, “efflux pumps”, “AmpC”, “beta-lactamase”, “carbapenemase”, “lipopolysaccharides”, “beta-lactam plasmids”, “gene flow”, “horizontal gene transfer”, “antimicrobial resistance”, “antibacterial agents”, “resistance genes”, “antibiotics adaptation”, “heavy metals”, “genomic evolution”, “pollution”, “stress responses”, “cryptic genes”, “evolution”, ”experimental evolution”, along with using “+”, “AND”, and “OR” for a specific search result. The identified articles were initially checked to determine their appropriateness to the subject, and all the relevant articles were read in detail. Clinical studies were excluded from the search results. Earlier literature reviews on the same topic were consulted to ensure key topics were not missed (Mahlen, 2011; Sanchez et al., 1997).

### Intrinsic resistance of *S. marcescens* efflux pumps and antimicrobial resistance

Efflux pumps are transmembrane proteins that regulate the extrusion of toxic components from the bacterial intracellular to the external environment (Blanco et al., 2016). Efflux pumps are able to recognize a diverse set of hazardous compounds including detergents, fatty acids, heavy metals, bile salts, dyes, and antibiotics (Marquez, 2005). Therefore, efflux pumps are among the most important contributors to intrinsic resistance phenotypes (Hernando-Amado et al., 2016). In addition, gene mutations might modify the efflux pump substrate specificity leading to acquired drug resistance (Blair et al., 2015).

Six families of efflux pumps have been described, from these the (i) ATP-binding cassette (ABC) transporters family, couples the energy derived from ATP hydrolysis to compound extrusion. In contrast, the (ii) major facilitator superfamily (MFS), (iii) small multidrug resistance (SMR), (iv) multidrug and toxic compound extrusion (MATE), (v) resistance/nodulation/cell division (RND) superfamily, and (vi) proteobacterial antimicrobial compound efflux (PACE) families operate as chemiosmotic coupling secondary active transporters powered by electrochemical ion gradients (Blanco et al., 2016; Du et al., 2018).

Genomic analysis revealed over 70 different efflux-associated genes broadly distributed among nosocomial and environmental *Serratia* isolates (Sandner-Miranda et al., 2018). This suggests that the origin and spread of a considerable set of these *Serratia* efflux genes ensued independently of exposure to antimicrobials, either in clinical or agricultural contexts.

Homologous genes of potential efflux systems have been found encoded within *S. marcescens* genomes (Iguchi et al., 2014; Sandner-Miranda et al., 2018) comprising representatives of five out of the six families: ABC, MATE, MFS, SMR, and RDN. The PACE family is the only one absent from sequenced *Serratia* strains (Table 1). In addition, representative members of the ABC, RDN, SMR and MFS efflux pumps have been implicated with multidrug resistance. Nonetheless, contribution of *S. marcescens* homologous genes of MATE efflux systems has not been evaluated yet.

**Table 1:** Characterized efflux systems of *S. marcescens*.

**Family**	**Efflux system**	**Antibiotic resistance**	**Xenobiotic resistance**	**Reference**
ABC	SmdAB	Norfloxacin Tetracycline	TPPCl DAPI	Matsuo et al. (2008)
	MacAB	Aminoglycosides Polymyxin	ND	Shirshikova et al. (2021)
RND	SdeXY	Tigecycline Tetracycline Ciprofloxacin Cefpirome Oxacillin Erythromycin Novobiocin	SDS Bile Salts	Hornsey et al. (2010) and Toba et al. (2019)
	SdeCDE	Novobiocin	ND	Begic & Worobec (2008)
	SdeAB	Fluoroquinolones Chloramphenicol Novobiocin	SDS EtBr	Kumar & Worobec (2005)
	SdeGH and SdePQ-OmsA	Novobiocin	Benzalkonium Chloride Triclosan SDS Deoxycholate EtBr	Toba et al. (2019)
	SdeIJ	ND	Benzalkonium Chloride	Toba et al. (2019)
SMR	SsmE	Norfloxacin	Acriflavine EtBr	Minato et al. (2008)
	SsmD	ND	ND	Minato et al. (2008)
MFS	SmfY	Norfloxacin	Benzalkonium Chloride Acriflavine EtBr TPPCl Methyl Viologen Hoechst 33342	Shahcheraghi et al. (2007)
	SmvA	ND	Chlorhexidine Octenidine Cationic Biocides	Wand et al. (2019)
	TetA	Tetracycline	ND	Thompson et al. (2007)

**Notes.**

TPPCl tetraphenylphosphonium chloride DAPI 4′,6-diamidino-2-phenylindole SDS sodium dodecyl sulphate EtBr Ethidium bromide ND Not determined

### ATP-driven efflux pumps (ABC)

The ABC transporters compose a diverse family of efflux pumps characterized for the presence of nucleotide-binding domains (NBDs) along to transmembrane domains (TMDs) (Dawson & Locher, 2006). The ATP binding/hydrolysis by the NBDs drives conformational changes that alternate between an inward-open and outward-open conformation to translocate substrates across the membrane (Choudhury et al., 2014). SmdAB (homolog to MdlAB of *E. coli*) was the first ABC efflux pump described in a clinical strain of *S. marcescens.* Mobilization of a plasmid expressing *mdAB* into the hyper-sensitive *E. coli* KAM32 conferred resistance to norfloxacin, tetracycline, tetraphenylphosphonium chloride (TPPCl) and 4′,6-diamidino-2-phenylindole (DAPI) (Matsuo et al., 2008).

MacAB (a canonical ABC-type efflux pump) has been linked to macrolide resistance in different *Enterobacteriaceae* members. However, in *S. marcescens* SM6 deletion of *macAB* did not impact to erythromycin sensitivity, instead the *macAB* strain was significantly more sensitive to aminoglycosides and to the CAPs colistin and polymyxin B (Shirshikova et al., 2021). Since *S. marcescens* is intrinsically resistant to this class of antimicrobials (as described below) the phenotype displayed by the *macAB* strain is intriguing. Nonetheless, in *Stenotrophomonas maltophilia* the MacAB homolog has also been implicated with extrusion of polymyxins, aminoglycosides and macrolides (Lin et al., 2014b). Notably, MacAB in *S. marcescens* SM6 was shown to be essential for survival during oxidative stress, also to contribute during biofilm formation and bacterial motility (Shirshikova et al., 2021), making this efflux pump an attractive target to reduce bacterial pathogenicity as well as antimicrobial resistance.

### Electrochemical gradient-driven efflux pumps (RND, SMR and MFS)

RND efflux pump family has been characterized widely in Gram negative bacteria; they consist of tripartite complexes composed by the inner membrane RND pump with 12 transmembrane regions; the periplasmic adaptor protein which connect the pump to the outer-membrane protein (OMP) responsible to assemble an OM conduit (Venter et al., 2015). The RND pump is responsible for drug selectivity and operates as an antiporter coupling drug/H^+^ transport. RND efflux pumps in *E. coli* incorporate TolC as the OMP (Nishino et al., 2003), however HasF is reported as the OMP mainly associated to the RND efflux pumps in *S. marcescens* (Hornsey et al., 2010).

The best characterized RND efflux pumps of *S*. *marcescens* are SdeXY (Chen et al., 2003a), SdeCDE, and SdeAB (Kumar & Worobec, 2005)*.* SdeXY was the first multidrug efflux pump characterized in this bacterium and it is described as a major contributor to *S. marcescens* intrinsic multidrug resistance (Chen et al., 2003b). Inactivation of *sdeXY* in the environmental strain NCTC10211 leads to an increased susceptibly to tigecycline, tetracycline, ciprofloxacin, and cefpirome (Hornsey et al., 2010). Similarly, deletion of *sdeXY* in *S. marcescens* Db10 conferred a significant reduction of MIC values to a broad spectrum of antimicrobial agents, including oxacillin, erythromycin, novobiocin, SDS, bile salts, among others (Toba et al., 2019). Moreover, up-regulation of *sdeXY* has been noticed in a clinical *S. marcescens* strain resistant to tigecycline (Hornsey et al., 2010). SdeAB efflux pump is considered to play a major role in *S*. *marcescens* multidrug resistance, it is implicated in extrusion of a wide range of compounds including fluoroquinolones, chloramphenicol, novobiocin, sodium dodecyl sulphate, and ethidium bromide (Kumar & Worobec, 2005). Gene deletion of *sdeB* in the clinical *S*. *marcescens* T-86 strain results in a multi-drug hyper-susceptibility phenotype. However, conflicting data indicate that the SdeAB substrate selectivity, expression, and contribution to drug resistance, is more likely strain specific (Begic & Worobec, 2008). Opposite to SdeXY and SdeAB, the SdeCDE efflux pump did not confer a multidrug resistance phenotype. SdeCDE activity has only been linked to novobiocin resistance (Begic & Worobec, 2008). A similar condition has been reported to the RND efflux pumps MdtABCD and MuxABC from *E*. *coli* and *P. aeruginosa*, respectively (Baranova & Nikaido, 2002; Mima et al., 2009).

Three additional RND efflux pumps SdeGH, SdeIJ, and SdePQ-OmsA have been identified in *S. marcescens* Db10 genome. SdeGH and SdePQ-OmsA were characterized as broad substrate specificities able to extrude benzalkonium chloride, triclosan, novobiocin, SDS, deoxycholate and ethidium bromide, whilst SdeIJ efflux pump only confers benzalkonium chloride resistance (Toba et al., 2019).

The SMR family is another efflux system conferring intrinsic resistance in *Serratia* genus. The SMR family is composed of small proteins (∼110 residues) which structure four transmembrane helices and operates as homodimers or heterodimers. SMR proteins extrude toxic compounds from the cytoplasm into the periplasmic space, consequently RND efflux pumps can complete the expulsion of toxic substances out of the cell (Tal & Schuldiner, 2009). In *E. coli* and *Salmonella,* SMR pumps confer resistance to a wide variety of antibiotics such as β-lactams, erythromycin, and tetracyclines, and to several quaternary ammonium compounds, such as benzalkonium chloride (Gadea et al., 2017). Nevertheless, in *S. marcescens* contribution of SMR to antibiotic resistance has been poorly investigated. SsmE was the first SMR efflux pump characterized in *S. marcescens*, it can extrude norfloxacin, acriflavine, and ethidium bromide (Minato et al., 2008) such substrate selectivity of SsmE is also conserved by its homologue EmrE, from *E. coli* (Minato et al., 2008). Another two SMR efflux pumps identified in *S. marcescens* are SsmD and SsmK, they are homologues to *E. coli* SugE and YdgE pumps, respectively, however they did not increase resistance to antimicrobial agents such as tetracycline, fluoroquinolones, aminoglycoside and topical antiseptics and their participation in the extrusion of xenobiotics has not been clarified (Minato et al., 2008).

On the other hand, the MFS family is the largest among the efflux pumps transporters, their members are well conserved in all phyla including bacteria, fungi, plants, and mammals (Pao, Paulsen & Saier Jr, 1998). Most MFS proteins operate as monomeric units (from 400–600 residues) with 12–14 transmembrane helices. In Gram negative bacteria the MFS transporters can be found as single proteins within the inner membrane also as part of tripartite efflux pumps (similar to RND pumps) (Neuberger, Du & Luisi, 2018). In *S. marcescens* Db11 the MFS efflux SmfY, was shown to confer resistance to several compounds such as norfloxacin, benzalkonium chloride, acriflavine, ethidium bromide, tetraphenylphosphonium chloride (TPPCl), methyl viologen and Hoechst 33342 (Shahcheraghi et al., 2007). SmvA (an SmfY homologous) encoded by *K. pneumoniae* and other *Enterobacteriaceae* (*e.g.*, *Salmonella*, *Citrobacter*, and *Enterobacter*) has been also shown to promote efflux of chlorhexidine, octenidine and other cationic biocides Wand et al., 2019.

In the environmental isolate *S. marcescens* FMC 1-23-O the MFS TetA was identified as a tetracycline resistant determinant (Thompson et al., 2007). The tetracycline-responsive repressor TetR is found encoded adjacently to *tetA*. In absence of stimuli TetR operates as a *tetA* repressor, however when there is intracellular tetracycline, antibiotic binding by TetR reduces its affinity for DNA, relieving *tetA* expression (Thompson et al., 2007). *S. marcescens* encodes some other MFS pumps yet to be characterized.

### Lipopolysaccharide modifications

*S. marcescens* as a Gram negative bacterium shows a low permeability due to the presence of its outer cell membrane. This structure confers a high rigidity that slows the passive diffusion of hydrophobic antibiotics like CAPs, which are amphiphilic short peptides produced by different species (including humans), that are able to interact and disrupt microbial membranes. Among the better characterized are found the group of polymyxins produced by *Paenibacillus polymyxa* (Velkov et al., 2016): polymyxin A and polymyxin E (called colistin) are used in clinical practices to treat extensively drug resistant Gram negative bacteria when other less toxic antibiotics (like carbapenems) are not an option (Tamma et al., 2022). CAPs interact with the lipopolysaccharide (LPS) molecule, displacing and disrupting Mg^2+^ cross bridges between anionic LPS molecules in the outer leaflet of the outer membrane, leading to cell envelope destabilization and cell death.

LPS is composed by lipid A, a core oligosaccharide (OS), and the O-antigen (O-Ag). Chemical modifications in the lipid A or into the core OS by adding positively charged substituents is associated with bacterial resistance to CAPs (Baron et al., 2016; Olaitan, Morand & Rolain, 2014b; Peschel, 2002). Particularly, LPS modification with 4-amino-4-*deoxy*-l-arabinose (L-Ara4N) moiety confers a net positive charge to bacterial surface which significantly reduces its affinity for polymyxins. In *S. marcescens* l- Ara4N is added to the lipid A, but also to the terminal d-*glycero*-d-*talo*-2-octulosonic acid (Ko) of the core OS (Vinogradov et al., 2006). LPS modification in *S. marcescens* occurs naturally making this bacterium intrinsically resistant to CAPs. Biosynthesis and addition of L-Ara4N to LPS requires the coordinated activation of *arnBCADTEF* operon, whose products mediate the synthesis and transfer of L-Ara4N to lipid A and core OS. Deletion of *arnB* and *arnC* genes in *S. marcescens* provoke a 1024-fold reduction on the MIC for polymyxin B (Lin et al., 2014a).

Expression of *S. marcescens arn* operon mainly responds to the two-component system (TCS) PhoP/PhoQ*.* Activation of PhoQ can be triggered through different stimuli including low Mg^2+^, acidic pH or polymyxin B. Accordingly, PhoQ becomes autophosphorylated then it transfers the phosphate moiety to PhoP, the phosphorylated PhoP binds directly to *S. marcescens arn* promoter operating as a positive transcriptional regulator (Barchiesi et al., 2012). Conservation of the PhoP binding site within the *arn* promoter across *Serratia* species suggest an important role of the PhoP/PhoQ-regulated *arn* pathway and LPS modification in this genus.

*S. marcescens arn* operon is also under the control of a second TCS, PmrA/PmrB. The activation of PmrA/PmrB system depends on a small protein called PmrD which is activated by the PhoP/PhoQ system. Thus, PmrD is a connector protein that links both regulatory systems, PhoP-PhoQ and PmrA-PmrB. Phosphorylated PmrA also recognizes *arn* promoter region and promotes RNA polymerase recruitment and transcription of the *arn* operon (Gunn et al., 1998).

The *S. marcescens phoP/phoQ* operon is positively auto-regulated, phosphorylated PhoP stimulates its expression by recognition of a regulatory site within *phoP*, however in absence of stimuli, *phoPQ* operon is expressed at basal levels from a constitutive promoter. In addition, the small regulatory transmembrane protein MgrB operates as a negative regulator of the PhoP/PhoQ system, disruption of *mgrB* result in *arn overexpression* (Olaitan et al., 2014a). Since deletion of regulatory proteins or TCS encoding genes retains low levels of *arn* expression, is considered that some other mechanisms are contributing to *arn* regulation in *Serratia*.

### Chromosomally encoded antibiotic modifying enzymes

In *S. marcescens* like other *Enterobacterales*, resistance to β-lactams is mainly related to production of penicilloyl-serine transferases commonly referred as β-lactamases, including AmpC-type cephalosporinase, extended-spectrum β-lactamases (ESBL, with activity against third generation cephalosporins and aztreonam), and carbapenemases.

The chromosomally encoded Ambler class C β-lactamase, AmpC, is invariably distributed among *S. marcescens* sequenced isolates, its activity is linked to inducible resistance to all penicillins as well as third generation cephalosporins (Jacoby, 2009; Joris et al., 1986). Despite the low affinity and hydrolytic capability against carbapenems (even at high AmpC protein levels) the combination of porin loss (OmpF) along with AmpC over production was reported to confer resistance against meropenem in *S. marcescens* urine isolates from Korea (Suh et al., 2010). Stability of *S. marcescens ampC* transcript is conferred by an extended 5′ untranslated region of 126 nucleotides adopting a stem-loop structure (Mahlen et al., 2003).

The *ampC* expression is induced by some β-lactams through accumulation of cytosolic peptidoglycan catabolites, they displace UDP-acetylmuramic acid peptides from AmpR impairing its activity as transcriptional repressor (Vadlamani et al., 2015). This signaling cascade is also emulated by mutations in the N-acetylmuramyl-alanine amidase AmpD or in the membrane-bound permease AmpG (Schmidtke & Hanson, 2006).

The *ampC* regulatory cluster (*ampD, ampE, ampR,* and *ampG*) is found ubiquitously among *S. marcescens* isolates. Nevertheless, in *S. marcescens* mutations leading to *ampC* derepression are estimated to occur *in-vitro* at rates ∼60-fold lower compared to *Citrobacter freundii* or *Enterobacter* species (Kohlmann, Bähr & Gatermann, 2018). Accordingly, Tamma et al. (2013) tested over 300 isolates by two phenotypic methods, determined that only 15% of *S. marcescens* isolates were positive for high-level of AmpC production compared to 38% of *Enterobacter* spp. isolates. In this scenario, Choi et al. (2007) showed that 7% of *S. marcescens* isolates developed resistance to extended spectrum cephalosporins during on patient drug therapy. While an other study from the same medical center revealed that none of 113 patients with *Serratia* infections (also treated with cephalosporins) developed antibiotic resistance (Choi et al., 2008). In contrast, emergence of *E. cloacae* resistance during therapy with extended-spectrum cephalosporins has been documented in 8% (Choi et al., 2008), and 19% (Chow et al., 1991) of the isolates.

In addition, some mutations leading to AmpC broader activity have been described in certain *S. marcescens* strains. These include the amino acid substitution S220Y located at the omega loop, it maps at the bottom of the entrance to the β-lactamase active site and increases by about 100-fold the catalytic efficiency of the enzyme conferring bacterial resistance to ceftazidime (Hidri et al., 2005). A rare 12 nucleotide deletion in *ampC* leading to the loss of four residues from the H-10-helix was also found to confer significant hydrolysis to fourth generation cephalosporins, cefepime and cefpirome (Mammeri et al., 2004).

A mechanism for carbapenem-resistance is conferred by the chromosomal SME (*S*. *marcescens* enzyme), this enzyme is capable of hydrolyze penicillin, aztreonam, cephalosporins, and imipenem. Nonetheless, since first report of SME-1 forty years ago in London (Naas et al., 1994), *S. marcescens* isolates carrying this gene have been rarely reported, most of them in North America and United Kingdom (Carrer et al., 2008; Cayo et al., 2017; Gales et al., 2001; Poirel et al., 2007; Queenan et al., 2006; Queenan et al., 2000).

Regarding aminoglycoside resistance, it often arises after chemical modifications through the activity of aminoglycoside modifying enzymes (AME). Three subclasses of AME have been described: the acyl-coenzyme A-dependent N-acetyltransferases (AAC); the nucleoside triphosphate-dependent *O*-phosphotransferases (APH); and the nucleoside triphosphate-dependent O-nucleotidyltransferases (ANT) (Ramirez & Tolmasky, 2010). Despite genes *aac(6′)-Ic* and *aph(3′)* are commonly found within *S. marcescens* chromosome (Sandner-Miranda et al., 2018) most clinical isolates remain susceptible to this class of antibiotics. Accordingly, scarce mRNA levels of *aac(6′)-Ic* have been identified across *S. marcescens* strains and a large palindromic sequence overlapping the −35 region of *aac(6′)-Ic* may be responsible of such low expression levels (Shaw et al., 1992).

## Acquired resistance

### Plasmid-encoded β-lactamases

Horizontal gene transfer is a common event in prokaryotes, it allows acquisition of loci encoding potentially advantageous elements under certain conditions. In addition, integrative and conjugative elements seem to contribute to gene flow between quite distant bacterial taxa (Sheinman et al., 2021). Regardless of gene transfer mechanism β-lactamases encoding genes are among the most frequent ARG transferred between bacteria, these enzymes are divided into four classes: the class A, C, and D employ a serine residue for inactivation of the β-lactam (Lamotte-Brasseur et al., 1994), whereas class B enzymes are metallo- β-lactamases that require Zinc as a cofactor (Crowder, Spencer & Vila, 2006).

In *S. marcescens* and related *Enterobacterales* the class A, CTX-M-type (cefotaxime enzyme), SHV (sulfhydryl variable), and the KPC (*K. pneumoniae* carbapenemase), along with class D β-lactamases OXA (oxacillinases), represent ESBL rapidly spreading between clinical strains (Bolourchi et al., 2022; Miao et al., 2019). The first notifications on the prevalence of KPC in *S. marcescens* clinical isolates became available at the beginning of the twenty-first century in China, Greece, Brazil, and United States (Cai et al., 2008; Firmo et al., 2020; Sidjabat et al., 2009; Tsakris et al., 2010; Zhang et al., 2007). Remarkably, the majority of KPC-producing *S. marcescens* strains were isolated from patients in surgical intensive care unit (Ferreira et al., 2020), suggesting acquisition of KPC plasmid at the nosocomial environment.

Carbapenem resistant *S. marcescens* strains recently isolated in China have been reported to carry *bla*_KPC-3_ (Chen et al., 2022), and *bla*_KPC-2_ (Wang et al., 2022), on incompatibility (Inc) group plasmids: IncX8 and IncX6-like, respectively. Also, a set of four carbapenem resistant *S. marcescens* isolates from Iranians patients patients were found to carry the *bla*_OXA_-48a gene on a plasmid belonging to the mcr incompatibility group (Bolourchi et al., 2022). A related plasmid (IncL-pOXA-48a) was previously detected among carbapenem resistant *S. marcescens* strains from Spain (Perez-Viso et al., 2021).

A comprehensive pangenome analysis of *S. marcescens* isolates from 35 countries revealed a population structure comprising 12 lineages (Matteoli et al., 2021). From these, *bla*_KPC-2_ plasmid was found widely distributed across lineage Sm7 (one plasmid per genome). Lineage Sm9 showed the highest plasmid diversity (3.26 plasmids per genome on average), with some members of this clade encoding a class D β-lactamase (Matteoli et al., 2021). The mobilized colistin resistance gene *mcr-9* was also detected in three strains from lineages Sm9 and Sm12 suggesting that some strains might function as reservoirs of *mcr-9* plasmids.

Metallo-β-lactamases are found among the most concerning enzymes, they can hydrolyze the broadest spectrum of penicillins, and their activities are not suppressed by next-generation inhibitors, as avibactam or vaborbactam (Boyd et al., 2020). Members of this group of enzymes comprise the versatile carbapenemases IMP (imipenemase), VIM (Verona Integron-encoded metallo-β-lactamase), and NDM (New Delhi metallo- β-lactamase) they are frequently transmitted through plasmids or integrons, and their presence in *S. marcescens* strains has been sporadically documented all over the world (Gajdacs & Urban, 2019).

In 2013 and 2019 nosocomial outbreaks caused by VIM-producing *S. marcescens* strains were reported at Argentina and Italy, respectively (Iovene et al., 2019; Nastro et al., 2013), the latter with a fatality rate of 50% (Iovene et al., 2019). Also, an outbreak associated with a strain producing IMP-4 and VIM-2 was documented in 2018 in Egypt (Ghaith et al., 2018). In 2020, Toth et al. (2020) isolated a VIM-4 producing *S. marcescens* from a Hungarian patient with no history of carbapenem treatment. While in 2021 in China Huang et al. (2021) recovered a carbapenem-resistant strain from a patient with asymptomatic bacteriuria, the resistant phenotype was associated with production of IMP-4 from a conjugative plasmid. A multi-resistant *S. marcescens* isolate carrying both genes *bla*_IMP-26_ and *mcr-9* within the IncHI2/2A megaplasmid was isolated during 2022 in China (Zhong et al., 2022).

### Plasmid-encoded ribosomal methyltranferases and aminoglycoside modifying enzymes

Ribosomal protection through methylation of 16S rRNA has been described as a principal mechanism of aminoglycoside resistance. Activity of 16S rRNA methylases confer high levels of aminoglycoside resistance however, as mentioned before most *S. marcescens* isolates remain susceptible to aminoglycosides with resistance predominantly through acquired means. Accordingly, *S. marcescens* S-95, an isolate from Japan encoding a new 16S rRNA methylase referred as RmtB displayed an unusual high degree of resistance to several aminoglycosides including kanamycin, tobramycin, amikacin, arbekacin, gentamicin, sisomicin, and isepamicin (Doi et al., 2004). Gene *rmtB* was found within a nonconjugative large plasmid also carryng the right end of transposon Tn3, including TEM-1 (Doi et al., 2004). RmtB shares 82% identity with the plasmid-encoded methylase, RmtA from *P. aeruginosa*. Consequently, its assumed that both genes have been mobilized by horizontal gene transfer in these pathogens*.*

On the other hand, prevalence of *S. marcescens* strains carrying conjugative plasmids encoding the AME enzyme AAC(6′), was reported in amikacin-resistant strains from three different cities in the U.S. evidencing that chromosomal encoded AAC(6′)-1 commonly coexists with homologues enzymes encoded by plasmids (John Jr et al., 1982). In addition, a novel aminoglycoside resistant gene, *ant(3″)-Ii-aac(6′)-IId* encoding an enzyme catalyzing bifunctional activities (adenylation and acetylation), was found in a 60-kb conjugative plasmid in *S. marcescens* SCH909 isolated in Greece (Centron & Roy, 2002). In this ANT(3′)-Ii/AAC(6′)-IId enzyme, adenyltransferase domain is highly specific to streptomycin and spectinomycin while the acetyltransferase domain displays a broader substrate range (Kim et al., 2006). APH(3′)-VIa is the only APH enzyme described in *S. marcescens,* the gene *aph(3′)-VIa* was found in plasmid R478 with a prevalence of 20% of the *S. marcescens* isolates from an Argentinian hospital (Garcia et al., 1996; Gilmour et al., 2004).

## Conclusions

*S. marcescens* is an ubiquitous microorganism which has gained recognition as an important opportunistic pathogen during last decades. In clinical practice, clearance of *S. marcescens* infections is challenging, largely because the immunological features of it most common hosts (newborns and immunocompromised individuals), but also because the wide repertoire of intrinsic antibiotic resistance determinants and resilience capacity displayed by *S. marcescens* strains, which narrow drug therapy opportunities and treatment success.

In this review, we aimed to compile our current knowledge of the mechanisms associated with the multidrug resistance phenotype of *S. marcescens*. To highlight, intrinsic elements of the RND along with MFS efflux pumps seem to confer the greatest range activity against antibiotics and toxic compounds. The AmpC activity is also responsibly of resistance to different penicillins, while its up-regulation might confer extended spectrum cephalosporins resistance. Moreover, plasmid-encoded carbapenemases (OXA, IMP, VIM and KPC) are sporadically reported in nosocomial *S. marcescens* strains. Prevalence of these carbapenemases among multi-drug resistant pathogens is a major concern. Thus, their continuous monitoring is critical in order to prevent wider spread of the mobile elements encoding this enzymes. Future studies evaluating contribution of other *S. marcescens* efflux systems, including SMR and MATE efflux pumps, along with regulatory mechanisms promoting adaptive resistance in this bacterium and how it can interact synergistically with intrinsic and acquired mechanisms are warranted.

Neglected use of antibiotics is considered a main driver of bacterial resistance, nonetheless, for some ubiquitous species (as several *Serratia* genus members), their long coevolution with natural antibacterial compounds has derived in defined patterns of drug tolerance/resistance. We also highlight here the complexity to distinguish and define *S. marcescens* nosocomial from environmental strains, and its potential as a natural reservoir of antimicrobial resistance elements.

Finally, human activities as environmental pollution and climate change are indirect drivers and stressors that might contribute to the emergence of resistant phenotypes. Hence, to obtain genetic information about taxonomic relatives of opportunistic pathogens and modulation of their genomes during natural interactions or after niche alterations might aid to elaborate mitigation plans to prevent future pathogenic outbreaks.

## Additional Information and Declarations

### Data Availability

The following information was supplied regarding data availability: There is no data to publish; this article is a literature review.
